# Toward an Open-Access Global Database for Mapping, Control, and Surveillance of Neglected Tropical Diseases

**DOI:** 10.1371/journal.pntd.0001404

**Published:** 2011-12-13

**Authors:** Eveline Hürlimann, Nadine Schur, Konstantina Boutsika, Anna-Sofie Stensgaard, Maiti Laserna de Himpsl, Kathrin Ziegelbauer, Nassor Laizer, Lukas Camenzind, Aurelio Di Pasquale, Uwem F. Ekpo, Christopher Simoonga, Gabriel Mushinge, Christopher F. L. Saarnak, Jürg Utzinger, Thomas K. Kristensen, Penelope Vounatsou

**Affiliations:** 1 Department of Epidemiology and Public Health, Swiss Tropical and Public Health Institute, Basel, Switzerland; 2 University of Basel, Basel, Switzerland; 3 Department of Biology, Center for Macroecology, Evolution and Climate, University of Copenhagen, Copenhagen, Denmark; 4 Department of Veterinary Disease Biology, DBL-Centre for Health Research and Development, University of Copenhagen, Frederiksberg, Denmark; 5 Informatics, Swiss Tropical and Public Health Institute, Basel, Switzerland; 6 The Open University of Tanzania, Dar es Salaam, United Republic of Tanzania; 7 Department of Biological Sciences, University of Agriculture, Abeokuta, Nigeria; 8 Department of Community Medicine, University of Zambia, Lusaka, Zambia; 9 Ministry of Health, Lusaka, Zambia; Case Western Reserve University School of Medicine, United States of America

## Abstract

**Background:**

After many years of general neglect, interest has grown and efforts came under way for the mapping, control, surveillance, and eventual elimination of neglected tropical diseases (NTDs). Disease risk estimates are a key feature to target control interventions, and serve as a benchmark for monitoring and evaluation. What is currently missing is a georeferenced global database for NTDs providing open-access to the available survey data that is constantly updated and can be utilized by researchers and disease control managers to support other relevant stakeholders. We describe the steps taken toward the development of such a database that can be employed for spatial disease risk modeling and control of NTDs.

**Methodology:**

With an emphasis on schistosomiasis in Africa, we systematically searched the literature (peer-reviewed journals and ‘grey literature’), contacted Ministries of Health and research institutions in schistosomiasis-endemic countries for location-specific prevalence data and survey details (e.g., study population, year of survey and diagnostic techniques). The data were extracted, georeferenced, and stored in a MySQL database with a web interface allowing free database access and data management.

**Principal Findings:**

At the beginning of 2011, our database contained more than 12,000 georeferenced schistosomiasis survey locations from 35 African countries available under http://www.gntd.org. Currently, the database is expanded to a global repository, including a host of other NTDs, e.g. soil-transmitted helminthiasis and leishmaniasis.

**Conclusions:**

An open-access, spatially explicit NTD database offers unique opportunities for disease risk modeling, targeting control interventions, disease monitoring, and surveillance. Moreover, it allows for detailed geostatistical analyses of disease distribution in space and time. With an initial focus on schistosomiasis in Africa, we demonstrate the proof-of-concept that the establishment and running of a global NTD database is feasible and should be expanded without delay.

## Author Summary

There is growing interest in the scientific community, health ministries, and other organizations to control and eventually eliminate neglected tropical diseases (NTDs). Control efforts require reliable maps of NTD distribution estimated from appropriate models and survey data on the number of infected people among those examined at a given location. This kind of data is often available in the literature as part of epidemiological studies. However, an open-access database compiling location-specific survey data does not yet exist. We address this problem through a systematic literature review, along with contacting ministries of health, and research institutions to obtain disease data, including details on diagnostic techniques, demographic characteristics of the surveyed individuals, and geographical coordinates. All data were entered into a database which is freely accessible via the Internet (http://www.gntd.org). In contrast to similar efforts of the Global Atlas of Helminth Infections (GAHI) project, the survey data are not only displayed in form of maps but all information can be browsed, based on different search criteria, and downloaded as Excel files for further analyses. At the beginning of 2011, the database included over 12,000 survey locations for schistosomiasis across Africa, and it is continuously updated to cover other NTDs globally.

## Introduction

More than half of the world's population is at risk of neglected tropical diseases (NTDs), and over 1 billion people are currently infected with one or several NTDs concurrently, with helminth infections showing the highest prevalence rates [1], [2]. Despite the life-long disabilities the NTDs might cause, they are less visible and receive lower priorities compared to, for example, the ‘big three’, that is malaria, tuberculosis, and HIV/AIDS [3], [4], because NTDs mainly affect the poorest and marginalized populations in the developing world [3], [5], [6]. Efforts are under way to control or even eliminate some of the NTDs of which the regular administration of anthelmintic drugs to at-risk populations – a strategy phrased ‘preventive chemotherapy’ – is a central feature [7]–[11].

There is a paucity of empirical estimates regarding the distribution of infection risk and burden of NTDs at the national, district, or sub-district level in most parts of the developing world [12]–[16]. Such information, however, is vital to plan and implement cost-effective and sustainable control interventions where no or only sketchy knowledge on the geographical disease distribution is available. There is a risk of missing high endemicity areas and distributing drugs to places which are not at highest priority, hence wasting human and financial resources. Consequently, integrated control efforts should be tailored to a given epidemiological setting [14].

The establishment of georeferenced databases is important to identify areas with no information on disease burden, to foster geographical modeling over time and space, and to control and monitor NTDs. In 1987 the bilingual (English and French) ‘Atlas of the Global Distribution of Schistosomiasis’ was published, which entailed country-specific maps of schistosomiasis distribution based on historical records, published reports, hospital-based data, and unpublished Ministry of Health (MoH) data [17]. While recent projects like the Global Atlas of Helminth Infections (GAHI; http://www.thiswormyworld.org) [18] and the Global Atlas of Trachoma (http://trachomaatlas.org) [19] offer maps on the estimated spatial distribution of soil-transmitted helminthiasis, schistosomiasis, and trachoma prevalence, they do not provide the underlying data for further in-depth analyses conducted by different research groups. An open-access global parasitological database for NTDs, which provides the actual data, is not available.

The Swiss Tropical and Public Health Institute (Swiss TPH) in Basel, Switzerland, together with partners from the University of Copenhagen, Denmark, and the University of Zambia (UNZA) in Lusaka, Zambia, were working together in a multidisciplinary project to enhance our understanding of schistosomiasis transmission (the CONTRAST project) [20], [21]. One of the CONTRAST goals was to create a data repository on location-specific schistosomiasis prevalence surveys in sub-Saharan Africa. In this manuscript, we describe the steps taken toward the development of such an open-access schistosomiasis database which is currently expanded to a global scale and to include other NTDs (e.g., soil-transmitted helminthiasis and leishmaniasis) and that can be constantly updated based on new publications and reports, as well as field data provided by contributors.

## Materials and Methods

### Guiding Framework

We selected schistosomiasis as the first disease to establish a proof-of-concept and populate our global NTD database. Indeed, schistosomiasis affects over 200 million people worldwide, with more than 95% concentrated in Africa. Both urinary schistosomiasis (caused by the blood fluke *Schistosoma haematobium*) and intestinal schistosomiasis (causative agents: *S. mansoni* and *S. intercalatum*) are endemic in Africa [22], [23].

**Figure 1:**
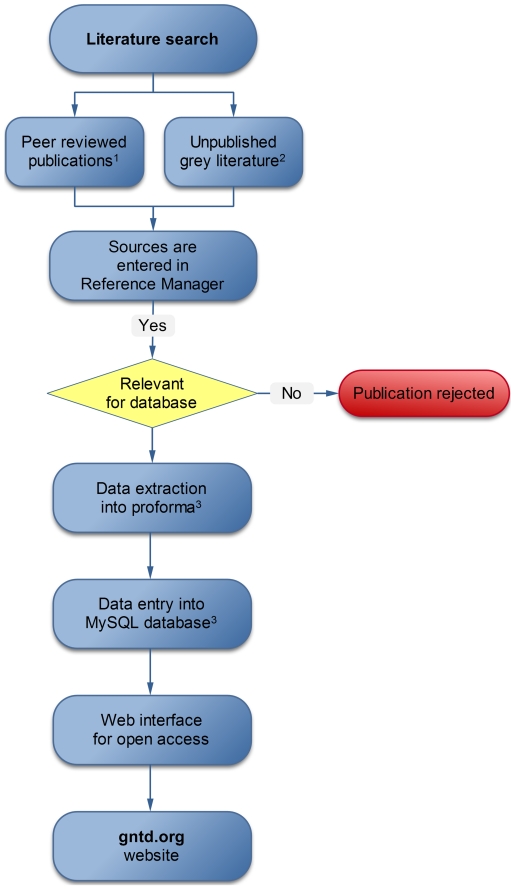
Flow-chart showing the steps used to assemble the GNTD database. 1. PubMed [24], ISI Web of Knowledge [25], African Journal Online (AJOL) [26], Institut de Recherche pour le Développement (IRD)-resources documentaries [28], WHO library archive [27], Doumenge et al. [17]; 2. Dissertations and theses in local universities or public health departments, ministry of health reports, other reports and personal communication. 3. Proforma and MySQL database include: (i) data source (authors); (ii) document type; (iii) location of the survey; (iv) area information (rural or urban); (v) coordinates (lat long in decimal degrees); (vi) method of the sample recruitment and diagnostic technique; (vii) description of survey (community-, school- or hospital-based); (viii) date of survey (month/year); and (ix) prevalence information (number of subjects examined and positive by age group and parasite species).

In order to obtain a large number of geographical locations to which prevalence data can be attached to our database, we conducted a systematic review. The specific steps of the process from identification of relevant surveys to data entry in the database, including various data sources, search criteria, data extraction and entry procedures, and quality control measures, are visualized in Figure 1, and will be described in more detail in the following sections.

### Data Sources

We systematically searched the following electronic databases with no restriction to date and language of publication: PubMed [24], ISI – Web of Knowledge [25], and African Journal Online [26]. Using specific search terms, we retrieved relevant peer-reviewed publications with an emphasis on schistosomiasis prevalence data in Africa.

The keywords applied for our literature search on schistosomiasis in the electronic databases, as well as the terms for the future search strategy on other NTDs, usually consists of species names and disease expressions often abbreviated and supplied with an asterisk in order not to miss out any results due to the variety of different spellings. The search strategy can be generalized as follows: *country name OR continent AND disease* (alternative spellings were included). These keywords were combined with names of African countries, whereas also alternative or former country names were considered to have our search strategy as broad as possible. This approach enabled us to save literature search results on a country-by-country basis.

Along with articles from peer-reviewed journals, reports from health institutions (e.g., World Health Organization (WHO) and the Office de la Recherche Scientifique et Technique d'Outre-Mer (ORSTOM)/Organisation de Coordination et de Coopération pour la Lutte contre les Grandes Endémies (OCCGE)) and doctoral theses (so-called ‘grey literature’) compose an important literature source for schistosomiasis prevalence data. Grey literature is often restricted to internal use or is not available in an electronic format. Publication databases available from WHO [27] and the Institute de la Recherche pour le Développement (IRD, formerly ORSTOM) [28] offer at least partial access to such documents. Additional grey literature included was obtained directly via site visits by team members to African universities and health research and development institutions. Another important source for survey data is the direct communication with local contacts, i.e., collaborators and partners from different African countries, individual researchers, and staff from ministries of health. The majority of entries that can be retrieved in the database were extracted from peer-reviewed journals (46%), however 30.5% of the data was obtained from personal communication with authors and 23.5% from grey literature. The latter was usually more extensive in terms of survey locations than the former sources. Since the key terms we used for our systematic review were mainly species and abbreviated disease names (e.g., ‘schisto*’ and ‘bilharz*’) that are not language specific, we also extracted and included reports written in languages other than English, including French (especially for West African countries), Portuguese, Italian, Dutch, Scandinavian and few in Russian and Chinese. Sources from literature and from personal communication were stored, labeled and managed with Reference Manager 11 [29].

Often, geographical information of the survey location was not given in the retrieved publications and reports (94%). Hence, we retrospectively georeferenced the locations. The majority of the coordinates was retrieved using the GEOnet Names Server (55%) [30], topographic or sketch maps (23%), and Google (14%) [31]. Personal communication with authors and local collaborators contributed another 5% of the retrospective geolocations, and only 3% were derived from other gazetteers and sources. Irrespective of the source of retrospective geolocation, we always mapped the coordinates in Google Maps to ensure that they are located in the study area and pointing to a human settlement. In general, we tried to adhere to the guidelines for georeferencing put forward by the MaNIS/HerpNEt/Ornis network to approach georeferecing in standardized manner [32].

### Data Extraction

All data sources obtained (literature, data from personal communication, and field visits) were screened for relevance by applying defined inclusion and exclusion criteria. Studies were included if they comprised prevalence data of schistosomiasis, identified either by school-based or community-based surveys. We accepted different study designs (cluster sampling, random sampling, stratified sampling, systematic sampling, etc.) as long as the reported findings could be considered as representative for the population or a specific sub-group of the population (e.g., school children, women, fishermen) in a given area. Along with schistosome prevalence data, a minimal set of information was collected, such as survey location (school, village, and administrative unit), date of survey, and number of individuals examined and found schistosome-positive (irrespective of sample size). In case additional survey-specific data were available, such as infection status according to age and sex, or intermediate host snail species (i.e., *Bulinus* spp. for *S. haematobium* and *Biomphalaria* spp. for *S. mansoni*), such information was tagged, as it might be of relevance for subsequent data extraction.

Hospital-based investigations, case-control studies, drug efficacy studies, and clinical trials, as well as reports on disease infection among travelers, military personnel, expatriates, nomads, and other displaced or migrating populations were excluded from the database in order to avoid non-representative samples (e.g., individuals with symptoms or disease-related morbidity) were excluded. Thus, the data in the database reflect the actual spatial distribution of the disease at a given time point. In case baseline prevalence data were reported in the aforementioned study types, or if former migrant populations settled down and the given survey location was clearly defined, data were included. Although having taken these precautionary steps, the database might still include prevalence data influenced by migration, since mobility and migration patterns of the rural population in sub-Saharan Africa are quite common [33], [34]. Based on our exclusion criteria, we rejected more than 70% of the articles retrieved from the literature search.

Once a source was identified as relevant, the data were extracted following a standard protocol with emphasize on (i) the source of disease data such as authorship, journal, publication date, etc.; (ii) description of the parasitological survey specifying the country, the survey date (year, month, season), and the type of survey (community- or school-based); (iii) survey location reported at the highest spatial resolution available; and (iv) parasitological survey data. If relevant source included malacological data, details on snail survey methods used, snail species collected, and infection rate of the Planorbidae were also extracted.

The Kato-Katz technique for *S. mansoni* and urine filtration for *S. haematobium* diagnosis are often considered as ‘gold’ standard methods [35]. If prevalence data were reported by different diagnostic methods, we only recorded in the database the results of the test with highest sensitivity and specificity. We applied the following ranking of diagnostic methods: (i) ‘gold’ standard; (ii) direct methods such as detection of eggs in urine/stool; and (iii) any other method such as antigen detection.

### Database System

The data are stored and managed in a MySQL [36] relational database with a web-interface built in hypertext preprocessor (PHP) [37]. The process from prevalence data extraction to database entry is schematically depicted in Figure 1.

The database consists of six tables corresponding to the sections of data extraction. The system architecture supports two types of users: the administrators and the end-users [38]. Registered administrators can enter new data, edit, or delete existing entries under their username and password. In addition, administrators can temporarily mask confidential data as requested by authors contributing specific data. Then a summary measure is presented instead with the contact details of the data owner to enable direct communication between researchers. Users can search all records using different selection criteria, e.g., country, document category, disease, and journal. The user part was designed to fulfill the most common queries, e.g., all recorded data for a specific parasite species in a given country or region within a specified period. The user will be able to download all information stored in the database matching different search criteria in an Excel file through an export function.

### Data Quality

To guarantee and improve data quality, the following measures have been taken. A first quality check is performed after data entry in the electronic database. For example, data extracted by assistants are always double-checked against the original source of information before becoming open-access, while data entries of senior staff are checked randomly. Data sent by contributors are inspected for completeness (e.g., in terms of study year and diagnostic technique), precise calculations (e.g., prevalence), and for correctness of coordinate information if provided. Additionally, we routinely screen the database for specific errors, i.e., by mapping survey locations and counterchecking whether the points are plotted in the expected area, by summarizing prevalence data per location and survey date to check for duplicate records, by testing for entry completeness.

Together with correctness of data extracted and entered, we also aim at the integrity of survey data. To further improve completeness of our database (e.g., date of surveys, disaggregated data) corresponding authors are contacted by e-mail asking for missing information. Approximately half of all reports had missing information, and so far we were able to get in touch with more than a third of the authors. Finally, missing coordinates for specific survey locations were obtained by re-checking additional maps and gazetteer sources, by communication with authors, and by employing global positioning system (GPS) databases created by collaborators during field visits for specific countries (i.e., Uganda, Zambia).

## Results

On 10 January 2011, our database contained 12,388 survey locations for schistosomiasis that are georeferenced from 35 African countries and 568 data points on intermediate host snails for 20 African countries, giving information on 25 different mollusk species. The database is constantly updated and subjected to quality control as the project moves along. Surveys are dated as early as 1900 and the historical references that are part of the Doumenge et al. (1987) [17] global schistosomiasis atlas are included by extracting data from the original source files. Since our main focus was on sub-Saharan Africa, the data currently included in the database covers all Western, Eastern, Middle and Southern African countries, according to UN Population Division classification [39]. Data extraction for Northern African countries is currently in progress. Survey coverage between countries shows considerable variation. Typically, larger numbers of survey locations were found in higher populated countries, but the amount of surveys also depends on existing national control or monitoring programs. In addition, temporal and spatial gaps in the survey distribution (as observed in Liberia, Rwanda, and Sierra Leone) might have occurred due to political instability and financial problems. The most widely used method for the diagnosis of intestinal schistosomiasis in the surveys that were fed into our repository is the Kato-Katz technique (76.7%, as single method or in combination). Stool concentration techniques accounted for a total of 13.3% (e.g., Ritchie/modified Ritchie technique (6.0%), concentration in ether solution (5.0%), merthiolate-iodine-formaline (MIF) concentration method (2.3%)) [40]. With regard to *S. haematobium* diagnosis, microscopic examination of urine after concentration (82.0%) such as urine filtration, urine centrifugation, and urine sedimentation, as well as reagent strip testing (12.8%) for the detection of blood in urine (i.e., microhematuria) or a combination of both approaches (2.3%) are most commonly employed.

**Figure 2:**
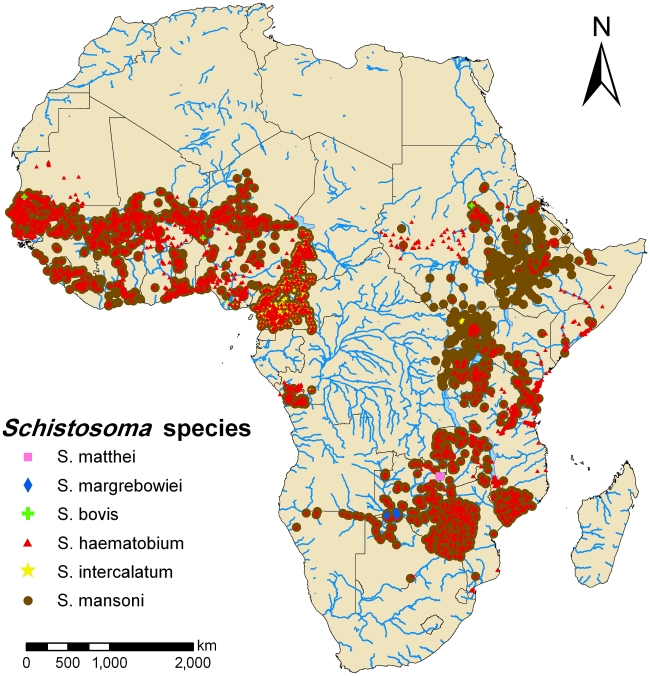
African map of schistosomiasis survey locations based on current progress of the GNTD database. Survey locations are represented by pink squares for *S. matthei*, blue diamonds for *S. margrebowiei*, yellow stars for *S. intercalatum*, green crosses for *S. bovis*, brown dots for *S. mansoni* and red triangles for *S. haematobium*. Surveys where subjects were screened for co-occurrence of multiple species are indicated with overlapping symbols.

Most of the surveys currently included in our database focus on school-aged children (70.1%), whereas less than a third (29.9%) of the surveys include all age groups. Furthermore, among the prevalence data of schistosomiasis collected, *S. haematobium* (54.4%) and *S. mansoni* (40.8%) were the most prevalent species. The third schistosome species parasitizing humans in Africa, *S. intercalatum* (4.8%), was only reported in surveys carried out in Cameroon and Nigeria, confirming that this species is restricted to some parts of West and Central Africa (Figure 2). Additionally, two zoonotic *Schistosoma* species were reported, namely *S. bovis* (0.02%) and *S. matthei* (0.01%), in the first cattle being the reservoir, while the latter is naturally affecting different antelope species (Table 1). Co-occurrence of multiple species was reported in 20.4% of the surveys, the majority of which (97.6%) was *S. mansoni*-*S. haematobium* co-occurrence. Currently, two schistosomiasis datasets in the GNTD database are confidential and about 100 datasets still await quality control. Hence, these data were masked and cannot yet be accessed by the database users.

**Table 1:** Number of *Schistosoma* spp. survey locations in the GNTD database in Africa stratified by country.

Countries	*S. mansoni*	*S. haema-tobium*	*S. inter-calatum*	*S. bovis*	*S. matthei*	*S. margre-bowiei*	Total
Angola	1	1	0	0	0	0	2
Benin	15	11	0	0	0	0	26
Botswana	34	26	0	0	0	0	60
Burkina Faso	55	257	0	0	0	0	312
Burundi	87	0	0	0	0	0	87
Cameroon	467	528	415	0	0	0	1410
Congo	2	86	0	0	0	0	88
Congo DRC	129	117	1	0	0	0	247
Côte d'Ivoire	229	225	0	0	0	0	454
Djibouti	1	0	0	0	0	0	1
Eritrea	10	8	0	0	0	0	18
Ethiopia	671	107	0	0	0	0	778
Gambia	5	56	0	0	0	0	61
Ghana	22	112	0	0	0	0	134
Guinea	37	38	0	0	0	0	75
Guinea-Bissau	0	38	0	0	0	0	38
Kenya	208	193	0	0	0	0	401
Liberia	93	120	0	0	0	0	213
Malawi	23	87	0	0	0	0	110
Mali	935	1007	0	0	0	0	1942
Mauritania	51	95	0	0	0	0	146
Mozambique	96	105	0	0	0	0	201
Namibia	32	32	0	0	0	4	68
Niger	237	858	0	1	0	0	1096
Nigeria	111	406	17	0	0	0	534
Rwanda	4	0	0	0	0	0	4
Senegal	238	699	0	1	0	0	938
Sierra Leone	37	64	0	0	0	0	101
Somalia	10	69	0	0	0	0	79
Sudan	202	179	0	1	0	0	382
Tanzania	292	576	0	0	0	0	868
Togo	80	77	0	0	0	0	157
Uganda	414	57	3	0	0	0	474
Zambia	183	311	0	0	1	0	495
Zimbabwe	169	219	0	0	0	0	388
**Total**	**5180**	**6764**	**436**	**3**	**1**	**4**	**12388**

Number of survey locations of *S. mansoni*, *S. haematobium*, *S. intercalatum*, *S. bovis*, *S. matthei* and *S. margrebowiei* as of 10 January 2011.

**Figure 3:**
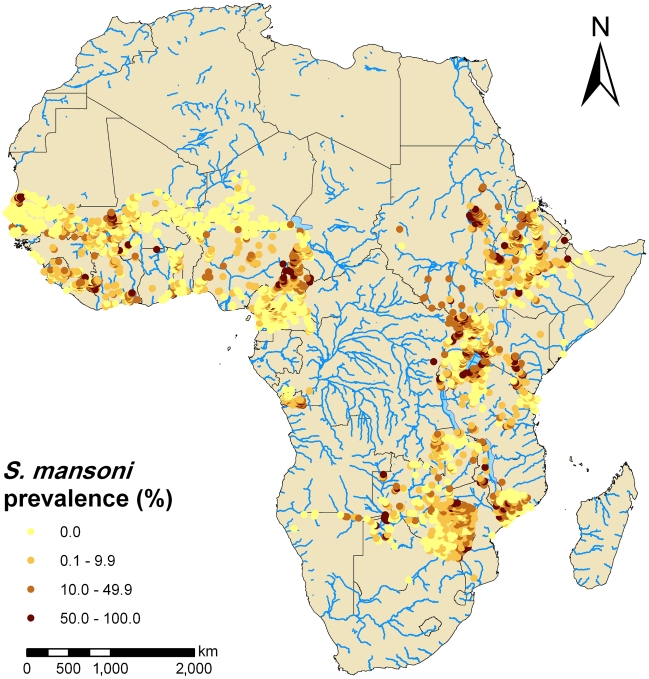
Observed prevalence of *S. mansoni* based on current progress of the GNTD database in Africa. The data included 4604 georeferenced survey locations. Prevalence equal to 0% in yellow dots, low infection rates (0.1–9.9%) in orange dots, moderate infection rates (10.0–49.9%) in light brown dots and high infection rates (≥50%) in brown dots. Cut-offs follow WHO recommendations [35].

**Figure 4:**
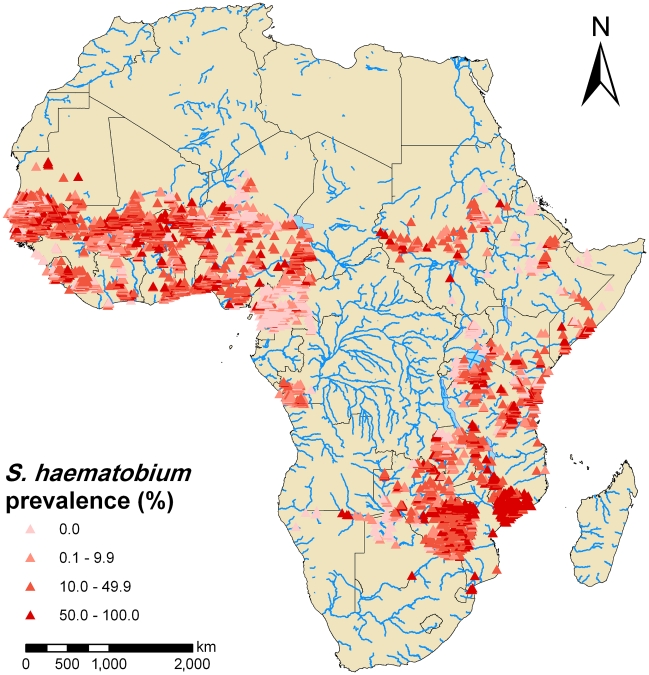
Observed prevalence of *S. haematobium* based on current progress of the GNTD database in Africa. The data included 5807 georeferenced survey locations. Prevalence equal to 0%, low infection rates (0.1–9.9%), moderate infection rates (10.0–49.9%) and high infection rates (≥50%) indicated by a red scale from light red to dark red. Cut-offs follow WHO recommendations [35].

The distributions of *S. mansoni* and *S. haematobium* are shown in Figures 3 and 4, respectively. The applied prevalence cut-offs of 10% and 50% were chosen based on WHO recommendations to distinguish between low (<10%), moderate (between 10 and 50%) and high (≥50%) endemicity communities [35]. The compiled survey data in the database suggest that *S. mansoni* predominates in East Africa, whereas *S. haematobium* prevalence is higher than *S. mansoni* in many African countries.

## Discussion

Data repositories are important tools for the development and validation of data-driven models to estimate the distribution and burden of NTDs, such as for malaria [41], [42]. Model-based predictions based on the compiled survey data will facilitate mapping of disease endemicity in areas without data and spatially explicit targeting of control interventions and long-term surveillance. With regard to NTDs, progress has been made for helminthic diseases [18] and trachoma [19]. The information included in a database helps to identify where current information is missing, request feedback from endemic countries, and initiate the collection of new data at those areas. Here, we described our efforts toward the establishment of an open-access database for NTDs. The database (http://www.gntd.or) allows for subsequent mapping of the observed survey data in order to identify high risk areas and to produce smooth risk maps, as exemplified by Schur et al. (2010) [43].

### Open-Access

The work presented here and the issue of open-access in relation to data, information sharing, and services, is not a new one. Indeed, we are following the successful implementations in different fields, e.g., open-access publishing (e.g., Public Library of Sciences (PLoS) and BioMed Central (BMC) journals), PubMed, genomic data [44]–[46], biodiversity [47], drug trial results [48], [49], and entertainment technologies [50].

With regard to epidemiological research, mapping disability, mortality, and disease burden due to infectious diseases, two recent open-access georeferenced epidemiological databases include the Mapping Malaria Risk in Africa (MARA), which is reporting malaria survey data in Africa dating back to 1900 [42], [51], and the Malaria Atlas Project (MAP) [41], which provides maps of raw and model-based estimates of malaria risk at a global scale and country level. Other examples are the WorldWide Antimalarial Resistance Network (WWARN) [52], [53], the MosquitoMap, a geospatially referenced clearinghouse for mosquito species collection records and distribution models [54], and the Disease Vectors Database [55], which is a georeferenced database on the presence of vector species of Chagas disease, dengue, leishmaniasis, and malaria. The GAHI project created a database of schistosomiasis and soil-transmitted helminthiasis survey data [13], [18], similar to our GNTD database, with the goal to provide open-access information on the global disease distribution and to highlight areas requiring mass drug administration. While the GAHI project focuses on mapping country-specific disease risk estimates, the GNTD database provides open-access to the mainly location-specific survey data. Free access to the data enables the users to conduct analyses for their own purposes. The existence of both databases offers the opportunity to join forces and to move forward in a unified way. As a first step it would be interesting to validate the two existing databases, align and harmonize them into a single comprehensive data repository, and discuss ways of harnessing synergies. Involvement of partners at WHO and other organizations will be essential.

### Limitations

Despite the benefits of free and public data repositories, data sharing is a challenge. Data owners may hesitate to provide their data, especially when they have not yet been published. However, confidential data can be masked through a special database feature as explained in the *Methods* section. As more and more data are included into the GNTD database, the current lack in the geographical extent of location-specific survey data across countries and regions will become less critical. Undoubtedly, a host of valuable information exists within countries, in the form of unpublished local archived sources. Efforts are ongoing to access this information with the help of our in-country scientific partners in ministries of health and research institutions by visiting the countries of interest to strengthen and further expand our global network of collaborators. Nevertheless, it is likely that there will remain significant areas with scarce data because no surveys have been conducted or data are not readily accessible or have been lost in the face of civil war, political unrest, or inappropriate archiving procedures. Such geographical lacks of survey data might be only known to local experts while the international community might not be aware.

Data from systematic literature searches or unpublished reports may contain different levels of reliability. For instance, snail identification is complex and without the guidance of experienced morphologists incorrect results may be reported. The quality of diagnostic methods must also be improved, for example through repeated stool and urine sampling over several consecutive days, since schistosome egg-output varies from one day to another. Unfortunately, only few surveys adopted such rigorous diagnosis due to generally limited financial and human resources [56], [57]. Furthermore, historical surveys differ in study design and are heterogeneous in terms of the age groups considered, the diagnostic methods applied, and the survey dates. Heterogeneity is also present in the way data are reported. For example in the past, numerous studies often aggregated their results at province or district level [58], [59], while currently information are frequently provided or shared at village or even individual level [60], [61]. All these points form important limitations of database compilations of epidemiological data. However, data are as limited as the sources from which they were derived. Developing standard NTD survey protocols, will enhance data comparability in the future [62].

Georeferencing historical surveys are not a straightforward undertaking. We have used a number of different sources to geolocate surveyed locations, the most common ones were described in the *Data Sources* section. However, several villages may have the same name within a single country. In such cases, information regarding the administrative boundaries of the village or its distance from nearby rivers, lakes, or towns is essential. A further complication is that administrative boundaries as well as region and district names may change over time. For instance, in Uganda, 23 new districts have been created in 2005 and 2006 [63].

In order to maintain high quality of the database, the entries are checked continually using systematic screening approaches as described in more detail in the *Methods* section. Additionally, we aim to further complement gaps (on date of survey, geographical coordinates, age group, number of people examined, etc.) and to obtain disaggregated survey data by contacting authors or collaborators, and by cross-checking new sources (maps, databases, and grey literature).

### Summary and Outlook

Our database is a global, freely-available, public, online resource, which hosts information pertaining to the distribution of NTDs. At present, the database contains more than 12,000 survey locations with emphasis on schistosomiasis prevalence data across Africa. It is currently expanded with information on soil-transmitted helminthiasis from Latin American and Southeast Asian countries. Our short-term goal is to extend the database from schistosomiasis to include other NTDs (i.e., ascariasis, hookworm disease, trichuriasis, lymphatic filariasis, onchocerciasis, and trachoma). Future versions of the database will supplement prevalence information from other NTDs (Buruli ulcer, Chagas disease, cysticercosis, dracunculiasis, leishmaniasis, leprosy, and human African trypanosomiasis). The approach for inclusion of further NTDs, as well as the search strategy that is going to be applied, will be the same as described in this article. We are aware that data on soil-transmitted helminthiasis is often given alongside intestinal schistosomiasis data and could have been extracted simultaneously. However, the database evolved from the CONTRAST project that focused on schistosomiasis. While screening for schistosomiasis, we labeled relevant references on other NTDs in our reference database, which will speed up future work steps, such as literature review and data extraction of relevant sources.

The structure of the database allows entering not only parasitological data, but also other attributes, like geospatially referenced data on the disease vectors. At present, our database has limited malacological survey information, and it does not include historical collections, however, we plan to add the georeferenced historical collection compiled by the Mandahl-Barth Centre for Biodiversity and Health in Copenhagen, Denmark, which holds information on about 7,000 georeferenced snail samples.

Our hope is to provide to scientists and policy makers, a user-friendly and useful platform which is continuously updated in order to facilitate data sharing, and retrieval of disease surveillance and epidemiological data. We welcome contributions from other researchers in possession of prevalence data from various NTDs. Users may contribute by download the template offered after registration and providing the required information. An administrator checks the data for quality and sends a confirmation e-mail before including the data in the database. Researchers who may not wish to share their data may only provide limited information about the data they possess (survey location, year, and amount of data) so that the database becomes a library of potential data sources. Furthermore, we plan to add an option for the GNTD database users to contact and interact with the contributors by providing a ‘send e-mail to contributor’ function.

Another immediate goal is to develop a web-based interface, which will combine raw disease data and spatial model-based estimates of disease burden at different geographical levels with country boundaries and geophysical information. The results will be accessed in geo-referenced kml format, which is displayed automatically on a Google Earth interface on the website [64]. This will allow users to obtain estimates of disease burden at different spatial resolutions (village, district, region, country, etc.) and to display model predictions including prediction uncertainties and raw data on the map.

A more distant option is to allow end-users to upload their own data, for instance regional and community-based health practitioners could directly upload disease prevalence to the MySQL database using hand-held smart phones with GPS functionality [65]. Success of the project will depend on active collaboration and contribution of researchers and disease control managers from around the world. We hope that our efforts will be recognized as a helpful tool contributing to the control and eventual elimination of NTDs.
